# Molecular Mechanisms of Persistence of Mutualistic Bacteria *Photorhabdus* in the Entomopathogenic Nematode Host

**DOI:** 10.1371/journal.pone.0013154

**Published:** 2010-10-05

**Authors:** Ruisheng An, Parwinder S. Grewal

**Affiliations:** Department of Entomology, The Ohio State University, Wooster, Ohio, United States of America; Indiana University, United States of America

## Abstract

Symbioses between microbes and animals are ubiquitous, yet little is known about the intricate mechanisms maintaining such associations. In an emerging mutualistic model system, insect-pathogenic bacteria *Photorhabdus* and their insect-parasitic nematode partner *Heterorhabditis*, we found that the bacteria undergo major transcriptional reshaping in the nematode intestine. Besides general starvation mechanisms, the bacteria induce cellular acidification to slow down growth, switch to pentose phosphate pathway to overcome oxidative stress and nutrition limitation, and shed motility but develop biofilm to persist in the nematode intestine until being released into the insect hemolymph. These findings demonstrate how the symbiotic bacteria reduce their nutritional dependence on the enduring nematode partner to ensure successful transmission of the couple to the next insect host.

## Introduction

The importance of microbial symbioses with animals is increasingly being recognized as a major theme in biology and characterization of such associations promises to revolutionize the way we view the biotic world [1]. As one of the most diverse animals on earth, nematodes have adapted to nearly every ecological niche from marine to fresh water and from soils to animal tissues. In fact, nematodes parasitize all known animals including themselves [2], and association with bacteria has certainly played a part in this evolutionary feat. The association of insect-pathogenic bacteria, *Photorhabdus* and *Xenorhabdus* with insect-parasitic nematodes, *Heterorhabditis* and *Steinernema*, respectively has emerged as one of the best-developed systems in symbiosis [3]. Their associations share a common biological function, allowing the partnership to infect, kill, and reproduce within an insect host, during which the bacteria promote their own transmission among insects using the infective juvenile as a vector whereas the nematode uses the bacteria as food [4], [5], [6]. Prior to infection, the bacteria are well protected from the external environment in the intestine of infective juvenile nematodes which endure without feeding for months in search of a suitable insect host in the soil. However, nothing is known how the bacteria persist in the non-feeding enduring infective juveniles. Nutritional dependence of *X. nematophila* on *S. carpocapsae* infective juveniles has been documented. *X. nematophila* mutants defective in methionine, threonine, and paraaminobenzoic acid and pyridoxine fail to persist in *S. carpocapsae* [7], [8]. Further, axenic *S. carpocapsae* infective juveniles survive longer than colonized ones, indicating a fitness cost incurred by the nematode [9]. Thus, the nematode appears to invest in the symbiotic association as the bacteria play a key role in its parasitic journey through the insect host [10]. As the stored energy reserves of the non-feeding infective juveniles are limited [11], the bacteria must make adjustments to reduce their nutritional dependence on the nematode in order to enhance persistence of the partnership. Herein, we investigated gene expression of *Photorhabdus temperata* residing in the enduring infective juveniles of *H. bacteriophora* to determine bacterial survival strategies and performed mutagenesis analysis to evaluate the importance of selected genes in bacterial persistence in the nematode intestine.

## Results

### Bacterial genes differentially expressed in the nematode

**Figure 1:**
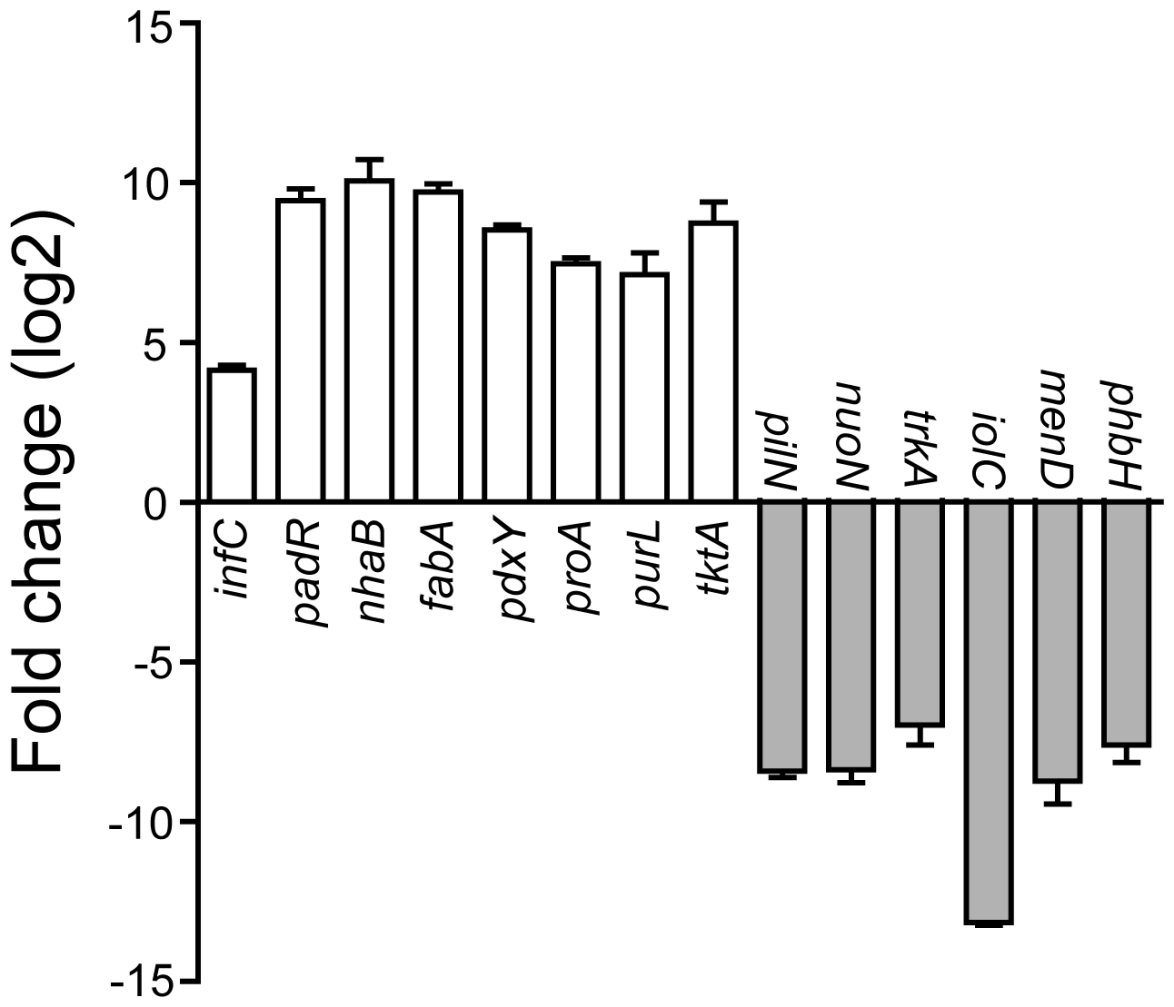
Quantitative real time PCR results showing fold changes in the expression of selected *Photorhabdus temperata* genes identified by SCOTS in the nematode *Heterorhabditis bacteriophora* infective juveniles compared to the *in vitro* culture.

*P. temperata* induced 50 and repressed 56 genes in the nematode partner compared to the *in vitro* culture (Table S1). Homology searches indicate that these identified genes are distributed in seven functional groups: cell surface structure, regulation, stress response, nucleic acid modification, transport, metabolism, and unknown transcripts (Fig. S1). Screening with the cDNA libraries prepared from bacteria grown *in vitro* under stationary-phase (starvation) conditions suggested that only about a half of the differentially expressed genes (26 induced and 23 repressed) were associated with starvation (Tables S1). Quantitative real-time PCR (qRT-PCR) performed on 14 randomly selected genes showed consistent results between the SCOTS and qRT-PCR assays. For example, both qRT-PCR and SCOTS showed an increased expression of *nhaB* and a decreased expression of *nuoN* in *P. temperata* in the nematode. Most tested genes displayed 6-12 fold changes in qRT-PCR assays (Fig. 1), suggesting a significant shift in bacterial gene expression in the nematode.

### Significance of the differentially expressed genes

**Figure 2:**
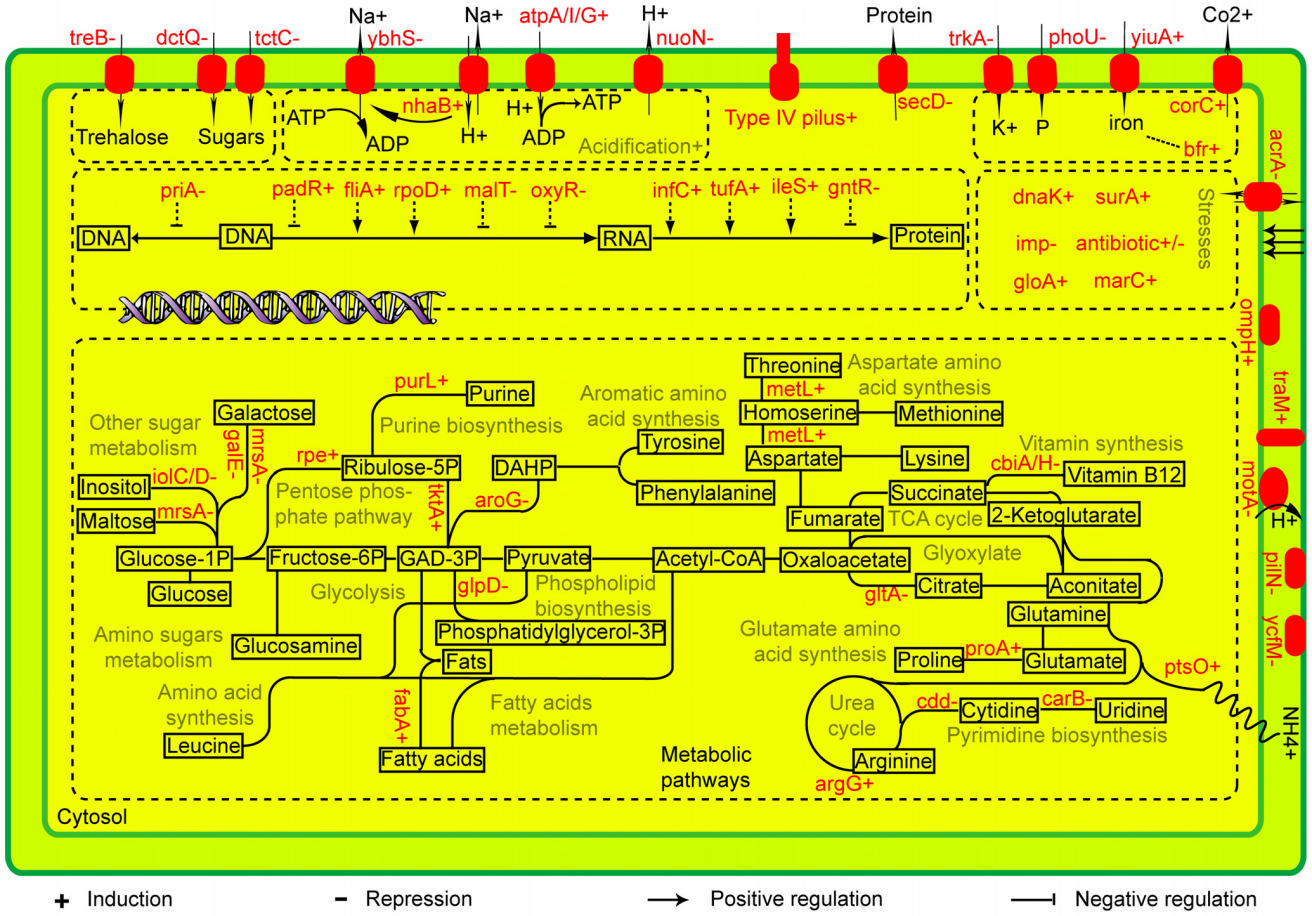
Conceptual molecular model illustrating comparative contributions of differentially regulated genes during symbiotic persistence of *Photorhabdus temperata* in *Heterorhabditis bacteriophora* infective juveniles.

Based on current functional understanding of the identified genes, a conceptual model was constructed to illustrate molecular processes involved in the persistence of *P. temperata* in its nematode partner (Fig. 2). With respect to metabolic adaptation, genes *iolC*, *iolD*, *galE* and *mrsA* by which carbon is converted to glucose for subsequent utilization were repressed. While three genes, *tktA*, *rpe* and *gntY*, involved in pentose phosphate pathway (PPP) were induced, a TCA cycle gene *gltA* and two genes *cbiA* and *cbiH* involved in vitamin B12 biosynthesis through TCA metabolites were repressed. Genes involved in amino acid metabolism were also found to be differentially regulated. For example, the gene *aroG* required for aromatic amino acid biosynthesis was repressed. Purine synthesis gene *purL* was induced, whereas pyrimidine synthesis genes *cdd* and *carB* were repressed. Further, a gene *nhaB* involved in proton uptake was induced but that for proton export *nuoN* was repressed. Genes corresponding to nutrient or growth factor uptake *treB*, *dctQ*, *tctC*, *trkA*, and *phoU* were also repressed. The gene required for cell motility *motA* was repressed while those for biofilm formation *fliA* and *traM* were induced. In addition, global changes in bacterial gene expression in the nematode partner were also reflected by the differential expression of genes such as *priA* and *padR* involved in replication and transcription processes, respectively (Fig. 2).

### Impact of acidification on bacterial growth and persistence

**Figure 3:**
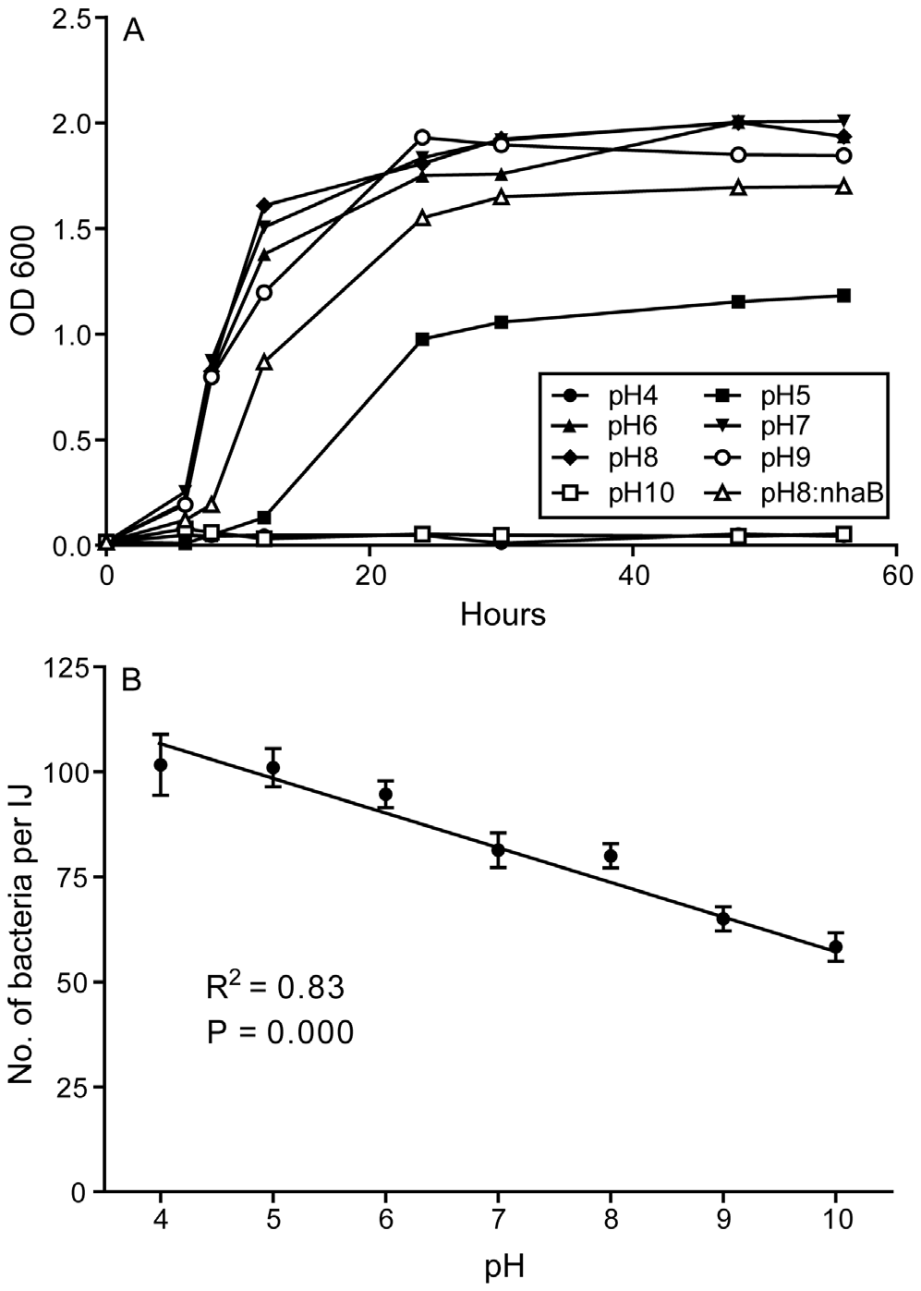
Impact of pH conditions on *Photorhabdus temperata* growth and viability. (A), Growth of *P. temperata* under pH range of 4–10 in the Brain Heart Infusion medium. ‘pH 8: nhaB’ denotes that *P. temperata* cells transformed with a plasmid bearing the intact *nhaB* gene grew in the Brain Heart Infusion medium at pH 8; (B), Relationship between pH and *P. temperata* survival in *Heterorhabditis bacteriophora* infective juveniles (IJ) exposed to different pH conditions for 14 days.

As demonstrated in the conceptual molecular model, induction of cellular acidification through differential expression of H^+^ transport genes appears to be one of the key physiological modifications made by *P. temperata* to persist in the enduring infective juveniles. Therefore, we determined the influence of pH on bacterial growth *in vitro* and on bacterial persistence in the infective juveniles. Bacterial growth was normal in the medium within a moderate pH range of 6–9, limited at pH 5, and no longer permitted at pH 4 or 10 (Fig. 3A). Further, bacterial cells transformed with the plasmid borne H^+^ import gene *nhaB* showed reduced growth in culture (Figs. 3A). This suggests that bacteria are capable of maintaining internal pH homeostasis by increasing proton uptake or minimizing its export. In addition, while survival of nematodes was not affected by the external pH conditions, acidic conditions prolonged survival of *P. temperata* cells in the infective juveniles, and the number of bacteria retained by the nematodes was correlated to the external pH (Fig. 3B).

### Symbiotic properties of the bacterial mutants

**Figure 4:**
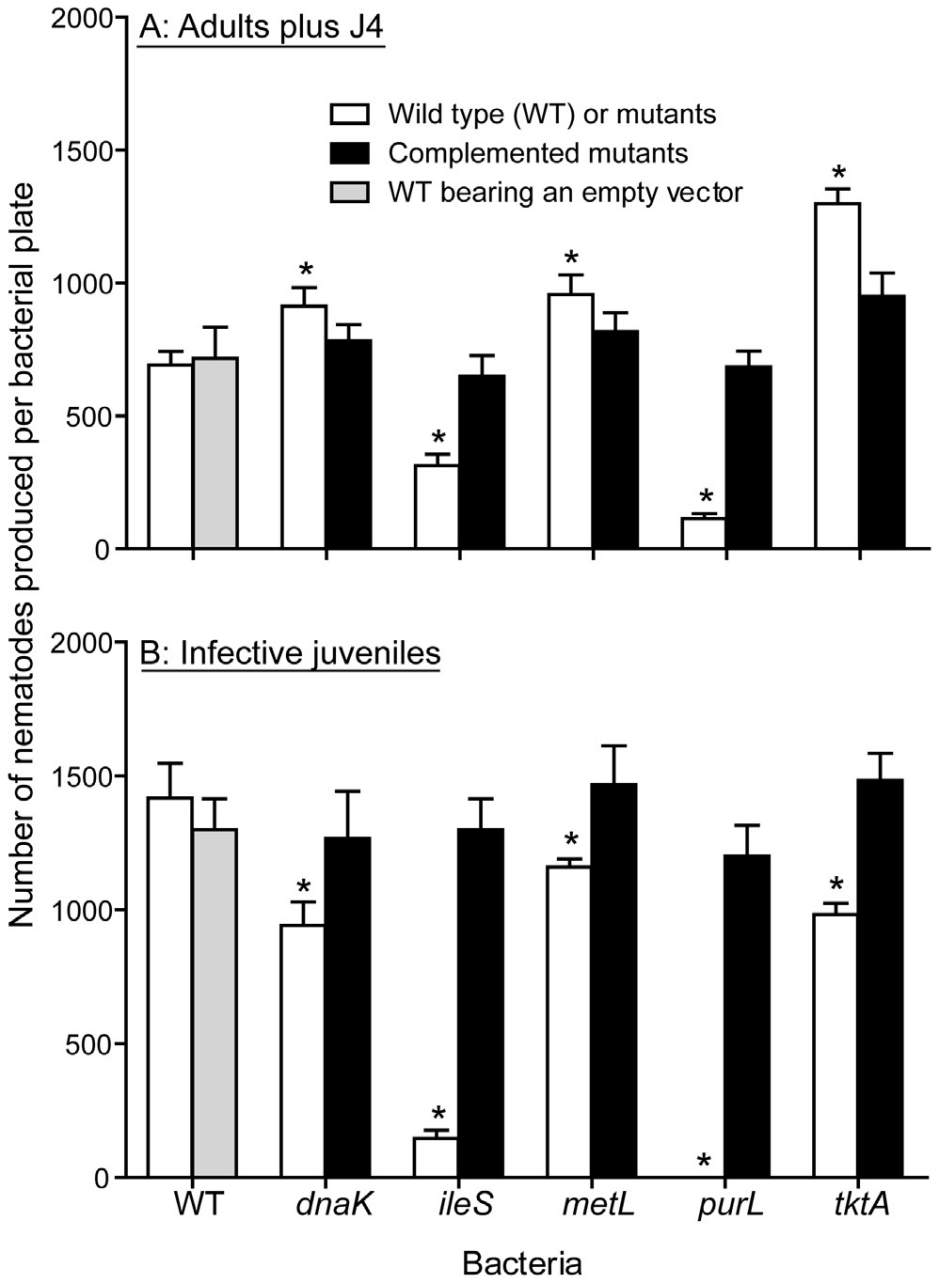
Reproduction of *Heterorhabditis bacteriophora* with *Photorhabdus temperata*. The results shown are average number (± SE) of *H. bacteriophora* adults and 4th stage juveniles (J4) (A) and infective juveniles (B) produced 14 days after inoculation of 100 bacteria-free nematode eggs on cholesterol agar plate seeded with wild-type (WT) bacteria, bacterial mutants or mutants complemented with plasmid borne respective genes. Wild-type bacteria containing the empty plasmid vector served as a control.

**Figure 5:**
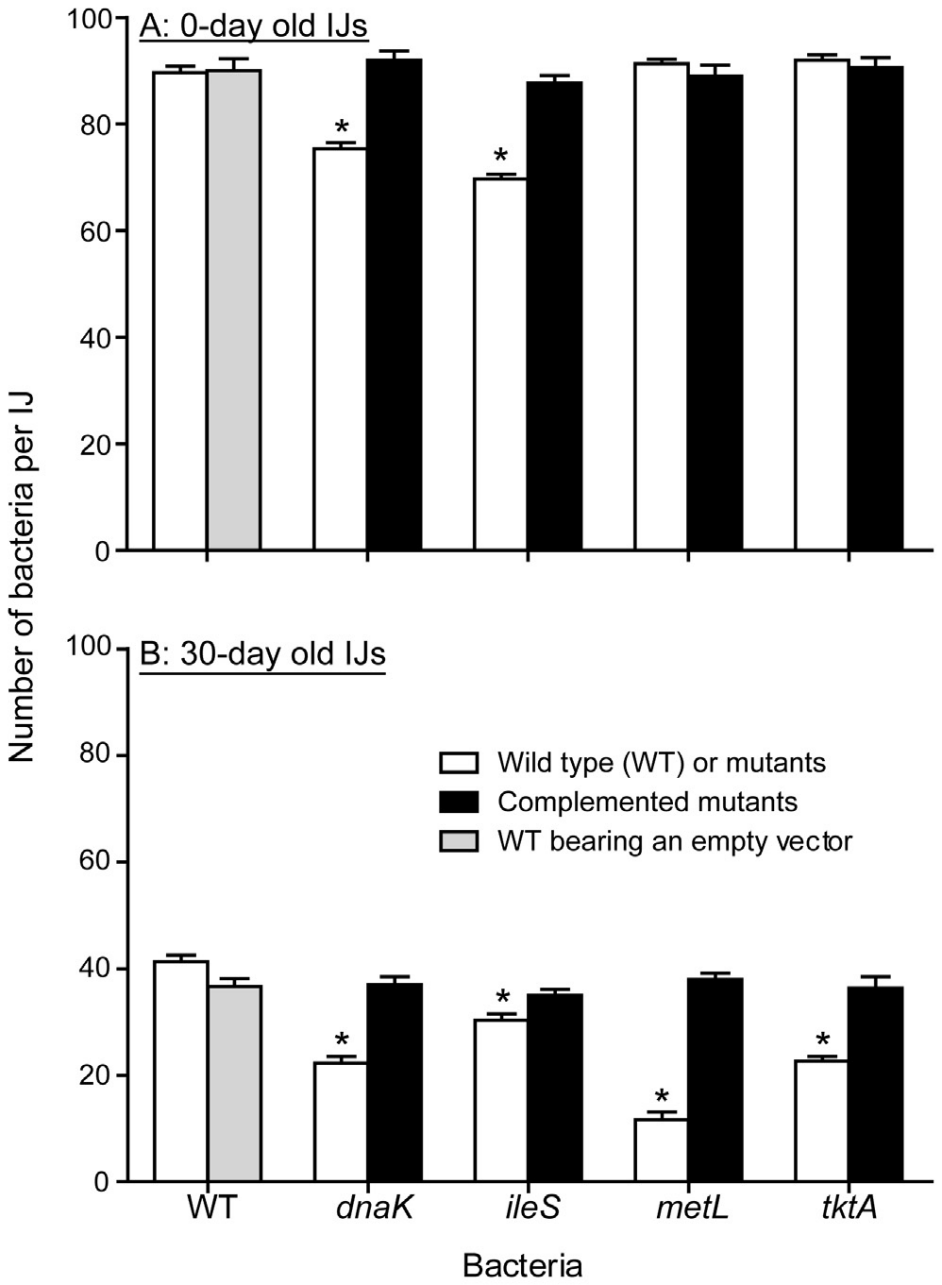
Mutualistic persistence of *Photorhabdus temperata* in *Heterorhabditis bacteriophora* infective juveniles. The results shown are average cell numbers (± SE) of mutant bacteria, mutant bacteria complemented with plasmid borne respective genes, or wild-type (WT) bacteria persisting in freshly-produced (A) and 30-day-old (B) infective juveniles. Wild-type bacteria containing the empty plasmid vector served as a control.

Five *P. temperata* mutants, termed as *dnaK^−^*, *ileS^−^*, *metL^−^*, *purL^−^* and *tktA^−^*, were created to investigate the importance of the selected genes in bacteria-nematode association. All these genes were found to be induced in the infective juveniles, and *dnaK* was also induced under *in vitro* stationary phase (starvation) conditions. These genes represent different cellular processes including a chaperone protein assisting in a number of cellular processes (*dnaK*), translation interpretation (*ileS*), amino acid metabolism (*metL*), purine and pyrimidine biosynthesis (*purL*), and pentose phosphate pathway (*tktA*). Phenotypes of these mutants were identical to the wild type in all tested properties including dye absorption, bioluminescence, colony morphology and growth rate, except their ability to support infective juvenile formation and colonization. Compared to the wild type, nematode reproduction was significantly lower with cells of *ileS^−^* and *purL^−^*, but higher with *dnaK^−^*, *metL^−^*, and *tktA^−^* (Fig. 4A). However, the numbers of infective juveniles produced on all mutants were lower than that on the wild type (Fig. 4B), and no infective juveniles were produced on *purL^−^*. Nematode development was initially observed with *purL^−^* but it quickly ceased. We observed that the level of infective juvenile colonization by *metL^−^* and *tktA^−^* in the freshly-produced infective juveniles was essentially the same as in the wild type, but was significantly lower in case of *dnaK^−^* and *ileS^−^* (Fig. 5A). Most importantly, the number of bacterial cells retained by 30-day old infective juveniles decreased more in mutants compared with the wild type (Fig. 5B). Mutants complemented with the plasmid carrying the respective genes were essentially the same as the wild type in all respects tested (Figs. 4 & 5).

## Discussion

Symbiotic microbes are ubiquitous in animal hosts, yet the study of symbiosis is still in its infancy [1]. Clearly, understanding of what and how microbial colonization and persistence factors are involved and regulated to bring about fitness outcomes is of fundamental importance. Our analyses of differentially expressed *P. temperata* genes reveal key molecular features reshaped by bacteria in mutualism with the nematode partner. We found that the bacteria switched to pentose phosphate pathway (PPP) in the nematode compared to TCA cycle in the culture and in the insect host [5]. Two PPP genes, *rpe* and *tktA*, were induced, and *gntY*, which facilitates gluconate metabolism via PPP, was found to be induced while *gntR*, whose product negatively regulates *gntY* expression, was repressed. With the induction of PPP, TCA cycle and glyoxylate pathways appear to be abandoned by *P. temperata* as indicated by the repression of citrate synthetase gene *gltA*. Also, genes *cbiA* and *cbiH* corresponding to the biosynthesis of vitamin B12 through TCA metabolites were repressed. Since vitamin B12 functions as a coenzyme in anaerobic fermentation of propanediol, ethanolamine and glycerol in most enteric bacteria [12], repression of *cbiA* and *cbiH* indicates the suppression of carbon metabolism in *P. temperata*. Further, repression of genes *glpD*, *iolC*, *iolD*, *mrsA* and *galE* by which other sources of carbon such as glycerol, inositol, maltose and galactose are converted to glucose for subsequent utilization suggests that alternative carbon sources may also not be available for *P. temperata* in *H. bacteriophora* infective juveniles. In fact, one of the possibilities for *P. temperata* to overcome the glucose starvation in the nematode may be the recycling of glucose-6-phosphate through the entry of PPP products fructose-6-phosphate and glyceraldehydes-3-phosphate into gluconeogenesis. Such recycling process is highly efficient as one molecule of glucose-6-phosphate is converted by 6 cycles of PPP and gluconeogenesis to 12 NADPH (36 ATP equivalents) [13].

The bacteria also appear to alter amino acid metabolism while residing inside the nematode. Induction of *metL* gene in *P. temperata*, which is known to be responsible for the synthesis of homoserine which serves as a precursor to threonine, lysine and methionine, reflects a growth-limiting concentration of these amino acids or an enhanced need for homoserine in the nematode as has been noted in *Salmonella* during infection of pigs [14]. Disruption of *metL* results in an increase in intracellular free lysine concentration during stationary phase in *Streptomyces clavuligerus* [15]. The fact that *metL* mutant supported nematode reproduction but had reduced persistence in the infective juveniles suggests that lysine may be helpful to *H. bacteriophora* growth and homoserine may be needed for *P. temperata* survival in the nematode. Aromatic amino acid biosynthesis in *P. temperata* was reduced as suggested by the repression of aromatic amino acid biosynthetic gene *aroG*. Further, differential expression of purine and pyrimidine biosynthesis genes *purL*, *cdd* and *carB* is likely required for bacteria-nematode interaction as *P. temperata purL* mutant was defective in supporting *H. bacteriophora* growth and completely inhibited infective juvenile formation. Considering the importance of purine and pyrimidine in lipopolysaccharide (LPS) properties, disability of *purL* mutant to support infective juvenile formation may be due to the LPS modification. LPS modification also seems to be important for colonization of *P. luminescens* since mutation of *pgbE1*, possibly responsible for modifying lipid A moiety of LPS, lost its ability to colonize nematode infective juveniles [16]. In fact, genetic modification of LPS has been thought to cause defective persistence of microbes in several symbiotic relationships including *Vibrio fisheri* and squids [17], [18], *Aeromonas* spp and leeches[19], and *Sinorhizobium fredii* and legumes [20].

The most intriguing process adopted by *P. temperata* inside the nematode appears to be the induction of intracellular acidification which may inhibit cell growth and protect cells from electrophile toxicity. We have confirmed that up-regulation of the proton uptake gene *nhaB* enables the bacteria to persist in alkaline conditions *in vitro*. Therefore, induction of proton importer gene and repression of proton exporter gene in *P. temperata* may cause intracellular acidification as has been noted in other bacteria [21], [22], [23]. Besides their contribution to proton influx, induction of *atpA*, *atpI* and *atpG* genes in *P. temperata* may indicate energy production through ATP synthesis [24]. In nematodes, ammonium constitutes 40–90% of non-protein nitrogen excreted from the body [25]. Induction of a urea cycle gene *argG* in *P. temperata* may indicate a possible mechanism to extrude excess nitrogen from cells [26] and regulate the ammonium pool. As alkaline conditions prevail inside the nematodes [25], differential expression of genes related to proton transport and ammonium pool regulation appear to be critical for bacterial persistence in the nematode host. Further, intracellular acidification has been suggested to negatively regulate expression of C4-dicarboxylate transporter gene *dctQ* [27]. Consistently, carboxylic transporter genes *dctQ* and *tctT* were found to be repressed in *P. temperata* inside the nematode. Because carboxylic transporters play important roles in the growth of anaerobic bacteria by contributing carbon and energy sources [27], repression of *dctQ* and *tctT* suggests limited carbon metabolism in *P. temperata*. Furthermore, repression of other transport systems such as *phoU* and *treB*, responsible for phosphate and trehalose import, respectively, also suggests reduced utilization of phosphate and trehalose leading to limited bacterial growth inside the nematode.

As physical contact between bacteria and the nematode is accomplished by the outer surface, bacterial cell envelope may be crucial for recognition and attachment of the bacteria to the nematode intestinal membrane. Our results indicate that biofilm is likely developed in *P. temperata*. As deletion of *motA* but not *fliA* in *E. coli* results in paralyzed motility with minimal defects in biofilm formation [28], [29], induction of *fliA* and repression of *motA* in *P. temperata* suggests that biofilm formation, but not cell motility, occurs in this species inside the nematode. These findings corroborate a recent study suggesting that motility is not required for symbiotic relationship between *P. luminescens* and *H. bacteriophora* [30]. As conjugative factors induce bacteria to form or enter in biofilm communities [31], induction of *traM*, encoding a conjugative DNA transfer factor, further support that biofilm may be developed in *P. temperata*.

Global changes in bacterial gene expression in the nematodes are also revealed by differential regulation of genes involved in replication, transcription and translation processes. Repression of *priA* in *P. temperata* may slow down its growth in the nematode host as *priA* mutation in *E. coli* results in decreased DNA replication [32] and bacterial growth [33]. Since *padR* encodes a negative transcriptional regulator of aromatic catabolism [34], induction of this gene in *P. temperata* may negatively regulate activity of bacterial aromatic catabolism in the nematode host, which is consistent with the repression of aromatic amino acids biosynthesis genes as discussed earlier. With reduced replication and transcription, translation level may also decrease. Because expression of *infC* is negatively auto-regulated at the level of translation [35], induction of this gene, however, indicates low level of translation in *P. temperata* in the nematode. Instead of promoting translation, induction of *tufA* gene may be responsible for tolerance to stresses [36] imposed by the nematode on *P. temperata*. Further, induction of *ileS*, with a function in linking amino acids to specific tRNAs, may reflect alternative needs for amino acids in bacterial translation processes. As *ileS* mutant was defective in supporting nematode growth and infective juvenile formation, induction of this gene in *P. temperata* suggests potential importance of amino acid Ile in nematode development. Finally, we speculate that differential expression of a number of transposase or helicase genes observed in this study may contribute to genetic variability for better adaptation and survival of the bacteria in response to the changed environment from active growth *in vitro* or in the insect hemolymph to reduced growth inside the nematodes.

In a previous study, we profiled *P. temperata* gene expression upon infection of an insect host relative to grown in artificial medium [5]. Some *P. temperata* genes found to be induced in the nematode in this study including *ompH*, *fliA*, *dnaK*, *surA*, *fabA* and *yncB* were also identified to be induced in the insect host, indicating certain genetic overlap between the bacterial pathogenicity and mutualism. However, many of the genes differentially expressed by the bacteria in the nematode are different, suggesting that the host has a huge role in determining the outcome of bacterial gene expression. Therefore, future work may aim at understanding how *Photorhabdus* bacteria interconnect the different interactions with their eukaryotic hosts by comparing gene expression between the bacteria in the infective juveniles versus those delivered into an insect host via the nematode.

In conclusion, this study profiles differentially regulated “symbiosis genes”, providing insights into the molecular mechanisms by which bacteria persist in their nematode vector. Future research on the novel bacterial genes found to be differentially expressed in the nematode intestine in this study may lead to the discovery of new bacterial persistence and colonization factors. Overall, the functional characterization of the identified symbiotic genes will enable further elucidation of molecular networks that allow for successful communication between microbes and animals.

## Materials and Methods

### Strains and maintenance

The infective juveniles of *H. bacteriophora* GPS11 were obtained from our liquid nitrogen frozen stock and maintained in the final instar *Galleria mellonella* larvae as described previously [37]. The bacterium *P. temperata* isolated from infective juveniles of *H. bacteriophora* GPS11 was routinely cultured in Brain Heart Infusion (BHI) media at 25°C unless otherwise stated.

### Selective capture of transcribed sequences (SCOTS)

SCOTS technique was used to profile gene expression of *P. temperata* in *H. bacteriophora* infective juveniles (Fig. S2). Total RNA samples were prepared from 24 h bacterial *in vitro* cultures or 10^6^ surface-sterilized freshly emerged infective juveniles using TRIzol reagent (Invitrogen) and treated with RNase-free DNase I (Ambion, Austin, TX). The *in vitro* and *in vivo* cDNAs were synthesized from the total RNA samples using random primers ST09N (5-ATC CAC CTA TCC CAG TAG GAG NNN NNN NNN) and ST189N (5-**GAC AGA TTC GCA CTT AAC CCT** NNN NNN NNN), respectively according to Froussard [38], after which they were amplified using the corresponding defined primers ST0 (5-ATC CAC CTA TCC CAG TAG GAG ) and ST18 (5-**GAC AGA TTC GCA CTT AAC CCT** ) and were normalized as described previously [5]. During normalization, a eukaryotic housekeeping 18S rRNA gene was used as a control to ensure that bacterial cDNAs were purified apart from the nematode cDNAs. For this purpose, presence of 18S rRNA gene in the *in vivo* cDNA populations before and after normalization was measured by PCR using 50 ng cDNA samples and primers 18SF (5-GGA ATT GAC GGA AGG GCA CCA) and 18SR (5-CCA GAC AAA TCG CTC CAC CAA C). Bacterial genes preferentially induced in the infective juveniles compared to the culture were enriched by subtractive hybridization of normalized *in vivo* cDNAs to the biotinylated bacterial genomic DNA that had been pre-hybridized with rDNA operon and normalized *in vitro* cDNAs. Vice versa, bacterial genes specifically repressed in the infective juveniles were enriched. Also, a prokaryotic housekeeping gene gyrase A (*gyrA*) was used as another control to ensure that only differentially expressed genes were captured after enrichment. The presence of *gyrA* in enriched cDNAs, and cDNAs before and after normalization was evaluated by PCR using primers gyrAF (5-ACG CGA CGG TGT ACC GGC TT) and gyrAR (5-GCC AGA GAA ATC ACC CCG GTC). The enriched bacterial cDNAs were cloned into an original TA cloning vector to construct libraries representing bacterial genes differentially expressed in the nematode relative to the *in vitro* culture.

### Bacterial genes differentially expressed in the nematode

A total of 720 clones from SCOTS constructed libraries were screened by southern blot hybridization as described previously [5], [39], [40] to identify bacterial genes differentially induced or repressed in the nematode. The individual clones from the enriched *in vivo* cDNA libraries that only hybridized to the probe made from normalized *in vivo* cDNAs and the individual clones from the enriched *in vitro* cDNA libraries that only hybridized to the probe made from normalized *in vitro* cDNAs were chosen for sequence analyses. Rarefaction analysis [41] was used to ensure the saturation of the coverage of the cDNA libraries for the identified genes [42], [43], [44], [45]. In case of low coverage estimated by rarefaction analysis, more clones would be selected from the enriched libraries for southern blot screening. As shown by the rarefaction curves (Fig. S3), saturation was achieved, indicating successful isolation of most representative genes from the enriched cDNA libraries. The functions of identified genes were assigned using BioCyc databases [46].

### Gene expression under starvation conditions

To identify genes expressed due to starvation, the identified transcripts were screened with SCOTS constructed stationary-phase cDNA library by southern dot-blot hybridization. For simulating anaerobic and starvation conditions that exist within the nematode, the saturated bacterial cultures were incubated for further 30 days at 25°C in sealed tubes without disturbance to reach a nutrient depleted stationary phase as described previously [47], [48]. The normalized stationary-phase cDNA library constructed by SCOTS was DIG-labeled and used to probe the identified transcripts by Southern blot hybridization as described above.

### qRT-PCR

To further validate and quantify the expression changes, qRT-PCR was performed in an IQ5 system (Bio-Rad) using QuantiTect SybrGreen PCR Kit (Qiagen) according to the manufacturer's instructions. Selection of representative genes depended on success of the primer design based on the identified sequences. All reactions were run in triplicate with three independent RNA samples and a no template negative control using the listed primers (Table S2). The relative fold change of each gene was calculated from ΔΔCt using 16s rRNA gene as an internal control.

### Influence of pH conditions on bacterial growth and persistence

As genes responsible for proton transport were identified to be differentially expressed in *P. temperata*, we evaluated impact of external pH conditions and internal expression of proton transport genes on bacterial growth *in vitro*, as well as influence of external pH conditions to bacterial persistence in the nematode. Overnight cultured wild-type and bacterial cells transformed with a pJB861 plasmid (National BioResource Project) carrying intact *nhaB* gene were diluted to an initial OD 600 of 0.03 and growth continued to approximately 60 h at 25°C with OD 600 monitored regularly. Wild-type cells were grown within a pH range of 4–10, and cells bearing intact *nhaB* gene were grown under pH 8. In addition, the numbers of bacterial cells in *H. bacteriophora* infective juveniles that were suspended in water for 14 days within a pH range of 4–10 at 25°C were assessed. One hundred infective juveniles surface-sterilized in 0.1% thimerosal solution and rinsed with sterile water were completely disrupted using an autoclaved micro pestle. The serial dilutions were spread on BHI agar, and bacterial colonies were counted following incubation at 25°C for 3 days. All assays were performed in triplicate using three independent cultures of the test bacterium. The data of bacterial growth at different pH conditions for each time point were subjected to the analysis of variance with significant differences tests at P = 0.05 using Minitab 15 (Minitab Inc.). Linear regression was used to analyze the relationship between external pH conditions and the bacterial persistence in the nematode. All the graphs were generated using Graphpad Prism 5.0 program (GraphPad software Inc.).

### Construction of bacterial mutants

Insertion-deletion mutations in five identified genes were constructed using fusion PCR strategy [49]. For each gene, three fragments F1 (the upstream of the target gene), *camR* (Chloramphenicol resistance gene) and F2 (the downstream of the target gene) were generated using primer pairs of P1 and P2, P3 and P4, and P5 and P6 (Table S3), respectively. The *camR* gene was amplified from the plasmid pAKCYC184 (BioLabs, New England) and the F1 and F2 gene fragments were amplified from *P. temperata* genomic DNA. The purified PCR products F1 and F2 were separately cloned into a pGEM-T easy vector (Promega), and then were re-amplified with a pair of T7/SP6 and overlap primers P5/P6. Approximately equal amounts of the three fragments F1, *camR* and F2 were mixed, and PCR assembled using the primers P1 and P6 which now served as the nested primers in the PCR reaction. The spliced fragment was restricted and ligated to the same enzyme digested suicide vector pKNG101 (BCCM/LMBP Plasmid Collection, Belgium). The ligature was introduced into wild-type *P. temperata* cells by conjugation with donor *E. coli* S17-1 lambda pir cells. The conjugant was selected on BHI agar containing 100 µg/ml ampicillin and 30 µg/ml chloramphenicol for the integration of the ligature into the *P. temperata* chromosome by a single homologous recombination event. The occurrence of a second crossing-over was then selected on BHI medium containing 5% sucrose, resulting in *P. temperata* mutants with stably integrated *camR* gene in the target locus. For complementation, the pJB861 plasmid containing the intact genes was transferred into the wild type and respective mutants by electroporation.

### Characterization of bacterial mutants

The phenotype of created mutants was compared to the wild-type *P. temperata* and to mutants reconstituted with the respective complementary plasmid (complementation of the mutant with the respective intact gene). The examined phenotypic traits performed in BHI medium included absorption of bromothymol blue dye, bioluminescence, colony morphology, and growth rate. Besides phenotypic characterization, effects of gene mutation on symbiotic interaction between the bacteria and the nematodes were assessed. Appropriate *P. temperata* cells were grown overnight at 25°C in 3 ml of BHI broth after which 50 µl were spread on cholesterol agar plate (1.5× nutrient broth, 1.5% agar and 10 µg/ml cholesterol) for incubation at 25°C overnight. Bacteria-free *H. bacteriophora* eggs were obtained from hermaphrodites using an alkaline lysis method as described by Lunau et al. [50]. Approximately 100 bacteria-free nematode eggs were propagated on each plate and incubated at 25°C. Nematode development was monitored daily under a microscope until the formation of infective juveniles. The numbers of 4th stage juveniles, adults, and infective juveniles were then counted. The numbers of bacterial cells in the infective juveniles were determined as described above. All assays were performed in triplicate, using three independent cultures of the test bacterium. The data were subjected to the analysis of variance with significant difference tests at P = 0.05.

## Supporting Information

Figure S1 Distribution of differentially expressed *Photorhabdus temperata* genes among various functional classes. The number of genes involved in cell surface, regulation, stress response, nucleic acid modification, transport, intracellular metabolism, and genes with unknown function or without similarity to known genes are presented.
(1.25 MB TIF)

Figure S2 Schematic presentation of the Selective Capture of Transcribed Sequences (SCOTS) technique followed by Southern blot analysis of SCOTS identified sequences. In panel A, normalized bacterial cDNAs were obtained directly from bacteria grown *in vitro* in the Brain Heart Infusion broth or *in vivo* in nematode infective juveniles (IJs). In panel B, cDNAs corresponding to genes preferentially induced or repressed in IJs relative to the broth were enriched by differential cDNA hybridization. The enriched cDNAs were transformed into a cloning vector to build the cDNA library. Cloned inserts were amplified by PCR, equally transferred to two nylon membranes, and probed with digoxigenin labeled normalized *in vivo* or *in vitro* cDNAs as described in Materials and Methods. The dots at the same position in the two arrays were loaded with the same amplicon of each individual clone from the enriched cDNA library, and the concentration of probes was standardized to be the same.
(3.77 MB TIF)

Figure S3 Rarefaction analysis curves demonstrating coverage of cDNA libraries for genes identified from bacteria *Photorhabdus temperata* during colonization of the nematode host *Heterorhabditis bacteriophora*.
(0.45 MB TIF)

Table S1 Differentially expressed *Photorhabdus temperata* genes in *Heterorhabditis bacteriophora* infective juveniles.
(0.07 MB PDF)

Table S2 Oligonucleotide sequences used for quantitative real-time PCR analyses.
(0.01 MB PDF)

Table S3 Oligonucleotide sequences used to generate *Photorhabdus temperata* mutant constructs in this study.
(0.01 MB PDF)
