# Supplementary figures and images for: Molecular Mechanisms of Persistence of Mutualistic Bacteria Photorhabdus in the Entomopathogenic Nematode Host
Source: PLoS One. 2010 Oct 5;5(10):e13154. doi: 10.1371/journal.pone.0013154 (PMC2950140; doi:10.1371/journal.pone.0013154)

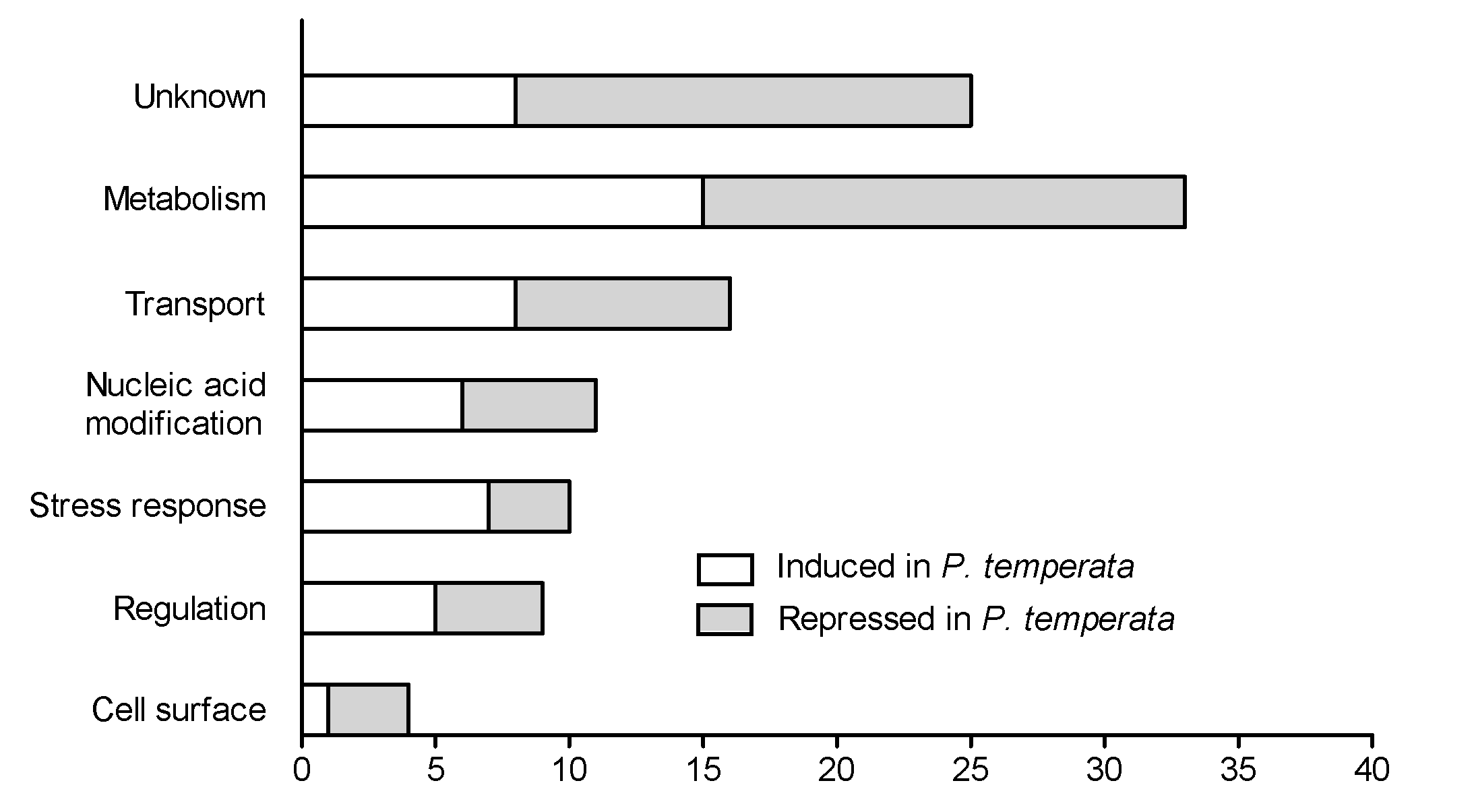

Supplement: Figure S1 — Distribution of differentially expressed Photorhabdus temperata genes among various functional classes. The number of genes involved in cell surface, regulation, stress response, nucleic acid modification, transport, intracellular metabolism, and genes with unknown function or without similarity to known genes are presented. (1.25 MB TIF) [file pone.0013154.s001.tif]

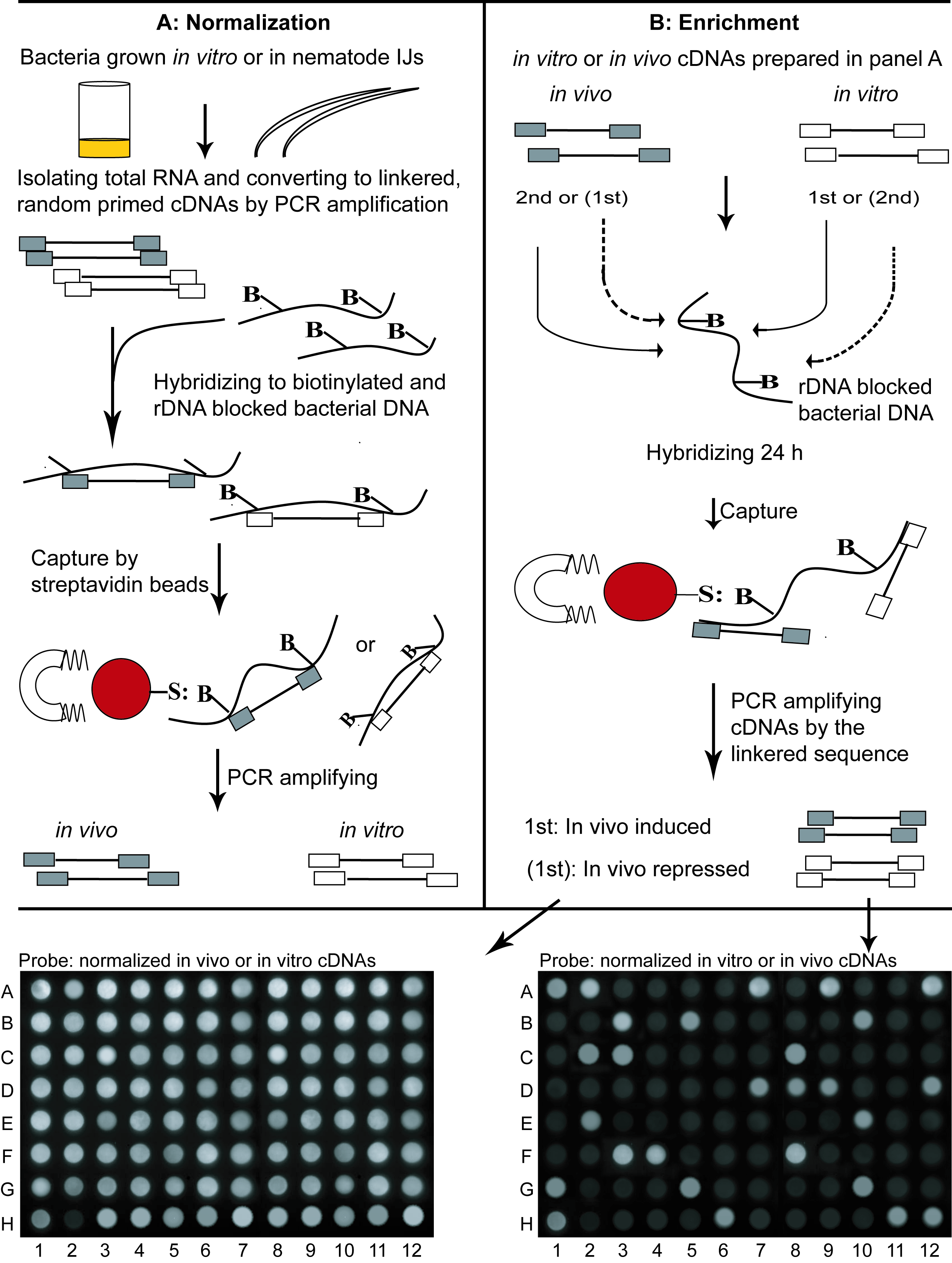

Supplement: Figure S2 — Schematic presentation of the Selective Capture of Transcribed Sequences (SCOTS) technique followed by Southern blot analysis of SCOTS identified sequences. In panel A, normalized bacterial cDNAs were obtained directly from bacteria grown in vitro in the Brain Heart Infusion broth or in vivo in nematode infective juveniles (IJs). In panel B, cDNAs corresponding to genes preferentially induced or repressed in IJs relative to the broth were enriched by differential cDNA hybridization. The enriched cDNAs were transformed into a cloning vector to build the cDNA library. Cloned inserts were amplified by PCR, equally transferred to two nylon membranes, and probed with digoxigenin labeled normalized in vivo or in vitro cDNAs as described in Materials and Methods. The dots at the same position in the two arrays were loaded with the same amplicon of each individual clone from the enriched cDNA library, and the concentration of probes was standardized to be the same. (3.77 MB TIF) [file pone.0013154.s002.tif]

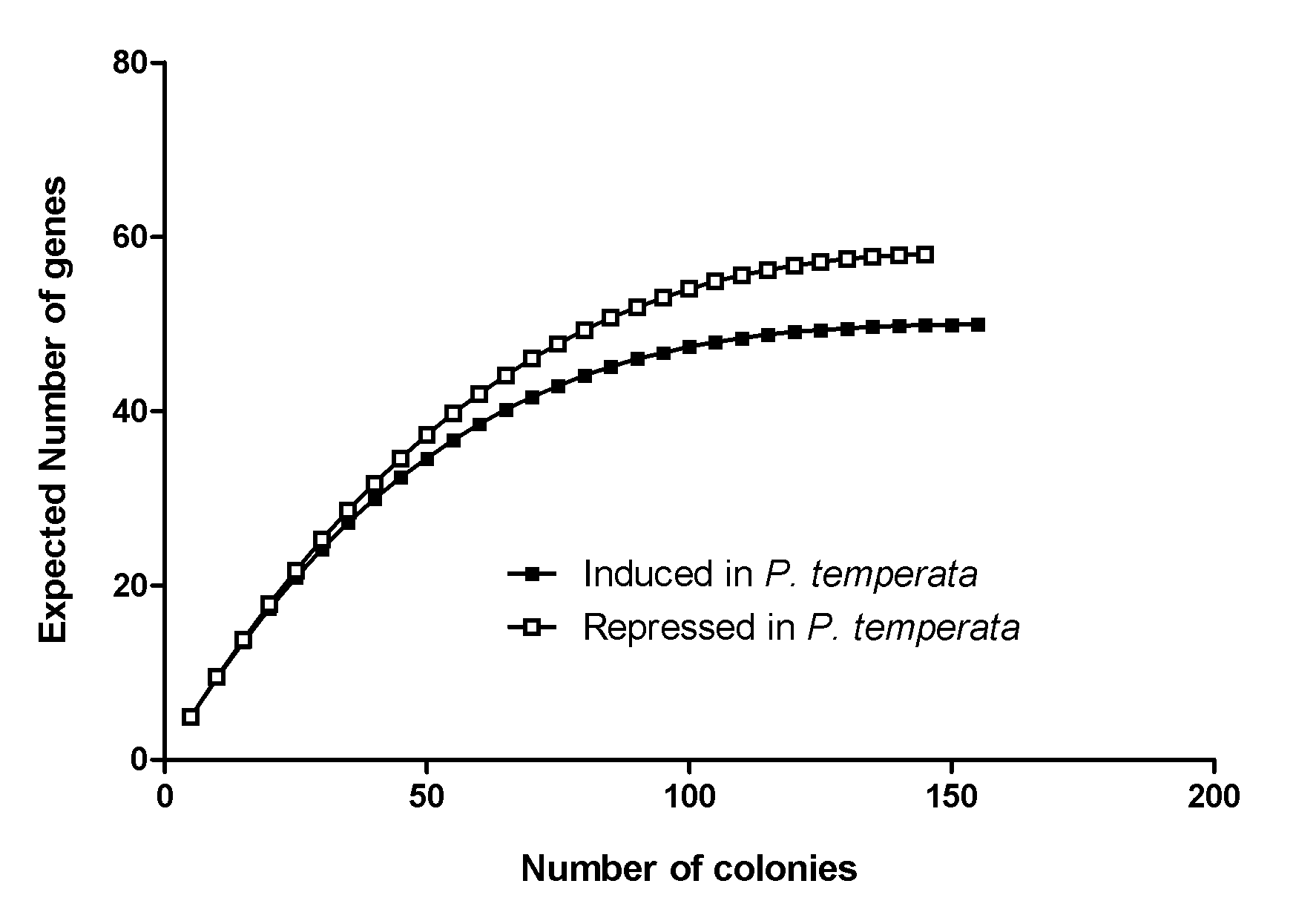

Supplement: Figure S3 — Rarefaction analysis curves demonstrating coverage of cDNA libraries for genes identified from bacteria Photorhabdus temperata during colonization of the nematode host Heterorhabditis bacteriophora. (0.45 MB TIF) [file pone.0013154.s003.tif]
