# Supplementary material for: Molecular Mechanisms of Persistence of Mutualistic Bacteria Photorhabdus in the Entomopathogenic Nematode Host
Source: PLoS One. 2010 Oct 5;5(10):e13154. doi: 10.1371/journal.pone.0013154 (PMC2950140; doi:10.1371/journal.pone.0013154)
Supplement: Table S1 — Differentially expressed Photorhabdus temperata genes in Heterorhabditis bacteriophora infective juveniles. (0.07 MB PDF) [file pone.0013154.s004.pdf]

Table S1. Differentially expressed *Photorhabdus temperata* genes in *Heterorhabditis bacteriophora* infective juveniles

Class	**Gene** -Possible function	Id/Sim%	Span	E-value
**Cell surface**	**<u>*ompH*</u> (+)** -Outer membrane protein skp precurson (*Photorhabdus luminescens*, plu0681)	91/98	165	1e-11
	***motA* (-)** -Chemotaxis protein (*Photorhabdus luminescens*, plu1849)	91/94	165	1e-27
	**<u>*pilN*</u> (-)** -Probable outer membrane lipoprotein (*P. luminescens*, plu1051)	100/100	182	2e-31
	**<u>*ycfM*</u> (-)** -Putative outer membrane lipoprotein (*P. luminescens*, plu2824)	93/100	139	7e-20
**Regulation**	***fliA* (+)** -Flagella biosynthesis RNA polymerase sigma factor (*P. luminescens*, plu1955)	98/100	150	8e-20
	**<u>*infC*</u> (+)** -Translation initiation factor IF-3 (*Erwinia carotovora*, ECA2421; *P. luminescens*, plu2668)	92/97	219	8e-31
	***padR* (+)** -Transcriptional regulator PadR-like family (*Xanthobacter autotrophicus*, Xaut_3967)	71/85	174	5e-18
	**<u>*rpoD*</u> (+)** -RNA polymerase sigma factor (*P. luminescens*, plu3979)	87/98	135	5e-15
	***tufA* (+)** -Translation elongation factor (*P. luminescens*, plu4730)	97/100	137	1e-23
	***gntR* (-)** -Repressor of gluconate operon (*P. luminescens*, plu0496)	87/91	169	9e-26
	***malT* (-)** -Positive regulator of mal operon (*P. luminescens*, plu0471)	93/100	129	5e-14
	**<u>*oxyR*</u> (-)** -Hydrogen peroxide-inducible genes activator (*P. luminescens*, plu4740)	82/88	153	5e-17
	**<u>*priA*</u> (-)** -Primosomal protein N', replication factor Y (*P. luminescens*, plu4759)	77/83	186	3e-22
**Stress response**	**<u>*bfr*</u> (+)** -Bacterioferritin (*P. luminescens*, plu4728)	98/100	186	1e-27
	**<u>*gloA*</u> (+)** -Glyoxalase/Bleomycin resistance protein/dioxygenase (*P. luminescens*, plu3746)	63/80	177	7e-17
	**<u>*dnaK*</u> (+)** -Chaperone (*P. luminescens*, plu0579)	100/100	141	5e-26
	**<u>*marC*</u> (+)** -Probable multiple antibiotic resistance protein (*P. luminescens*, plu2495)	90/90	180	1e-22
	**<u>*surA*</u> (+)** -Peptidyl-prolyl cis-trans isomerase (*P. luminescens*, plu0611)	90/93	195	3e-26
	**(+)** -Probable protein involved in antibiotic biosynthesis (*P.*	82/88	203	6e-30

	luminescens, plu3130)			
	**(+)** -Probable protein involved in antibiotic biosynthesis (*P. luminescens*, plu3263)	100/100	164	8e-32
	acrA **(-)** -Acriflavin resistance protein/Multidrug efflux protein (*P. luminescens*, plu3851)	75/76	194	5e-26
	imp **(-)** -Organic solvent tolerance protein precursor (*P. luminescens*, plu0612)	94/98	148	9e-29
	**(-)** -Probable peptide synthetase involved in antibiotic synthesis (*P. luminescens*, plu3123)	83/95	125	2e-16
**Nucleic acid modification**	***deaD*** **(+)** -Inducible ATP-independent RNA helicase (*P. luminescens*, plu4523)	100/100	174	1e-16
	orn **(+)** -Oligoribonuclease (*P. luminescens*, plu4595)	98/98	174	1e-24
	rep **(+)** -ABC-type DNA helicase (*P. luminescens*, plu4667)	100/100	171	5e-25
	ydaB **(+)** -Tail fibre protein (*P. luminescens*, plu1464)	73/79	168	4e-17
	**(+)** -ISSod6 transposase (*Alteromonas macleodii*, MADE_01489)	74/84	171	6e-16
	**(+)** -IS630-family transposase (*P. luminescens*, plu4076)	92/94	133	1e-16
	tnpA **(-)** -Putative ATP-binding IS21-family transposase (*Escherichia coli*, ECS88_4898)	78/90	144	2e-22
	uvrD **(-)** -UvrD-family helicase (*Escherichia coli*, EcE24377A_3362)	65/84	176	3e-17
	**(-)** -IS982-family transposase (*P. luminescens*, plu3299)	54/73	193	5e-19
	**(-)** -IS630-family transposase (*P. luminescens*, plu0310)	68/78	197	3e-14
	**(-)** -IS4-family transposase (*Shewanella* sp, Sputw3181_2039)	68/76	127	2e-15
**Transport**	***atpA*** **(+)** -ATP synthase alpha chain (*P. luminescens*, plu0042)	94/98	142	4e-26
	atpG **(+)** -ATP synthase subunit C (*P. luminescens*, plu0041)	100/100	183	2e-27
	atpI **(+)** -ATP synthase subunit I (*P. luminescens*, plu0047)	88/93	177	2e-11
	corC **(+)** -Mg^{2+}/Co^{2+} efflux protein (*P. luminescens*, plu1309)	98/98	132	3e-16
	nhaB **(+)** -Na+/H+ antiporter (*P. luminescens*, plu2563)	91/96	243	3e-26
	traM **(+)** -Tram protein (*Serratia entomophila*, AF135182)	76/88	126	5e-12
	yiuA **(+)** -Periplasmic siderophore binding protein of ABC transporter (*P. luminescens*, plu2853)	68/83	207	1e-20
	**(+)** -Probable type IV secretion protein (*P. luminescens*, plu0371)	98/98	150	2e-21
	dctQ **(-)** -Tripartite ATP-independent periplasmic (TRAP) C4-dicarboxylate transporter (*Vibrio vulnificus*, VPA1703; *P. luminescens*, plu0174)	50/67	138	2e-13

	**<u>*nuoN*</u> (-)** -NADH dehydrogenase subunit N (*P. luminescens*, plu3077)	82/94	297	8e-39
	**<u>*phoU*</u> (-)** -Phosphate transporter (*P. luminescens*, plu0218)	100/100	129	3e-19
	**<u>*secD*</u> (-)** -Protein-export protein (*P. luminescens*, plu3902)	95/97	154	6e-21
	***tctC* (-)** -Tricarboxylic transporter (*P. luminescens*, plu3181)	100/100	105	1e-12
	***treB* (-)** -PTS system, trehalose-permease IIBC component (*P. luminescens*, plu3288)	72/80	186	3e-22
	**<u>*trkA*</u> (-)** -K+ importer (*P. luminescens*, plu4671)	77/87	232	2e-32
	**<u>*ybhS*</u> (-)** -ABC-type sodium efflux transporter permease protein (*P. luminescens*, plu1507)	94/97	102	2e-5
**Metabolism**	***argG* (+)** -Argininosuccinate synthase (*P. luminescens*, plu4742)	87/94	141	3e-16
	**<u>*fabA*</u> (+)** -Beta-ketoacyl synthase (*Saccharopolyspora erythraea*, SACE_0023)	44/63	237	8e-15
	**<u>*gntY*</u> (+)** -Probable gluconate metabolism protein involved in iron-sulfur cluster biosynthesis (*P. luminescens*, plu0198)	94/100	159	5e-24
	***ileS* (+)** -Isoleucyl-tRNA synthetase (*P. luminescens*, plu0591)	87/100	159	6e-21
	***metL*(+)** -Aspartate kinase II/Homoserine dehydrogenase II (*P. luminescens*, plu4755)	100/100	156	1e-21
	**<u>*pdxY*</u> (+)** -Pyridoxine kinase (*P. luminescens*, plu2595)	94/95	234	3e-37
	***proA* (+)** -γ-glutamyl phosphate reductase (*P. luminescens*, plu1244)	91/95	240	5e-35
	***ptsO* (+)** -Phosphocarrier protein (*P. luminescens*, plu4045)	93/98	168	6e-21
	***purL* (+)** -Phosphoribosylformylglycinamidine synthase (*P. luminescens*, plu3317)	98/100	186	2e-27
	***rpe* (+)** -Ribulose-5-Phosphate 3-epimerase (*P. luminescens*, plu0086)	98/98	186	2e-28
	**<u>*sixA*</u> (+)** -Phosphohistidine phosphatase (*P. luminescens*, plu3199)	88/95	177	7e-22
	***tktA* (+)** -Transketolase (*P. luminescens*, plu0946)	100/100	213	3e-34
	***xpsD* (+)** -Peptide synthetase (*Xenorhabdus bovienii*, AF455810)	53/73	203	4e-18
	**<u>*yncA*</u> (+)** -Probable GCN5-related N-acetyltransferase (*P. luminescens*, plu4503)	76/84	147	1e-13
	**<u>*yncB*</u> (+)** -Probable Zn-dependent oxidoreductase (*Escherichia coli*, EFER_3231; *P. luminescens*, plu4072)	77/91	159	8e-18
	***aroG* (-)** -3-deoxy-D-arabino-heptulosonate 7-phosphate synthetase (*P. luminescens*, plu1470)	86/90	150	3e-16
	**<u>*carB*</u> (-)** -Carbamoylphosphate synthetase (*P. luminescens*, plu0604)	96/98	194	5e-34

	cbiA **(-)** -Cobyrinic acid a,c-diamide synthase (*P. luminescens*, plu2999)	98/100	177	5e-28
	cbiH **(-)** -Precorrin-3 methyltransferase (*P. luminescens*; plu2991)	90/93	233	5e-32
	cdd **(-)** -Cytidine deaminase (*Lysinibacillus sphaericus*, Bsph_0353)	64/73	130	5e-09
	clpA **(-)** -ATP-dependent Clp protease (*P. luminescens*, plu1594)	85/94	161	2e-22
	cpdB -2',3'-cyclic-nucleotide 2'-phosphodiesterase precursor (*P. luminescens*, plu3927)	87/91	146	2e-19
	elbB **(-)** -Enhancing lycopene biosynthesis protein 2 (SCRP-27A) (*P. luminescens*, plu4007)	96/98	162	7e-30
	galE **(-)** -UDP-glucose 4-epimerase (*P. luminescens*, plu4831)	85/91	151	5e-27
	glpD **(-)** -Aerobic glycerol-3-phosphate dehydrogenase (*P. luminescens*, plu0194)	64/75	156	1e-10
	gltA **(-)** -Citrate synthase (*Photorhabdus asymbiotica*, FM211057; *P. luminescens*, plu1425)	92/94	116	9e-18
	hslV **(-)** -ABC type protease (*P. luminescens*, plu4762)	95/95	144	1e-26
	iolC **(-)** -Putative myo-inositol carbohydrate kinase (*P. luminescens*, plu1801)	88/94	229	2e-37
	iolD **(-)** -Putative thiamine pyrophosphate-dependent myo-inositol carbohydrate kinase (*P. luminescens*, plu1805)	90/90	146	1e-26
	menD **(-)** -Menaquinone biosynthesis protein (*P. luminescens*, plu3073)	70/87	222	1e-24
	mrsA **(-)** -Phosphoglucomutase protein (*P. luminescens*, plu4533)	96/100	173	5e-30
	phbC **(-)** -Isochorismate synthase PhbC (*P. luminescens*, AY042783)	85/92	161	2e-24
	phbF **(-)** -Photobactin synthetase (*P. luminescens*, AY042783)	100/100	159	2e-24
	phbH **(-)** -Photobactin synthetase PhbH (*P. luminescens*, AY042783)	88/91	203	3e-35
	queF **(-)** -NADPH-dependent 7-cyano-7-deazaguanine reductase (*Erwinia tasmaniensis*, ETA_27310; *P. luminescens*, plu0662)	68/73	136	1e-09
**Unknown**	**(+)** -Hypothetical protein (*P. luminescens*, plu0801)	87/90	93	1e-6
	**(+)** -Hypothetical protein (*P. luminescens*, plu0830)	95/97	180	9e-25
	**(+)** -Hypothetical protein (*P. luminescens*, plu1160)	97/100	90	3e-8
	**(+)** -Hypothetical protein (*P. luminescens*, plu1774)	95/95	114	2e-11
	**(+)** -Hypothetical protein (*P. luminescens*, plu2035)	81/98	129	1e-13

	**(+)** -Unknown sequence flanked by plu3378 and plu3379 (*P. luminescens*)	96/96	159	1e-26
	**(+)** -Hypothetical protein (*P. luminescens*, plu3387)	93/97	174	2e-25
	**(+)** -No similarity to known genes			
	**(-)** -Hypothetical protein (*P. luminescens*, plu0428)	77/87	177	2e-26
	**(-)** -Hypothetical protein (*P. luminescens*, plu1582)	75/78	227	1e-22
	**(-)** -Hypothetical protein (*P. luminescens*, plu1927)	76/86	139	1e-18
	**(-)** -Hypothetical protein (*P. luminescens*, plu2339)	84/89	129	6e-14
	**(-)** -Hypothetical protein (*P. luminescens*, plu3186)	86/93	224	1e-07
	**(-)** -Hypothetical protein (*P. luminescens*, plu4553)	87/94	174	1e-23
	**(-)** -No similarity to known genes (3+4)			

Note: Up-regulation and down-regulation of genes are indicated by **(+)** and **(-)**, respectively. Genes underlined were also differentially regulated in response to *in vitro* stationary-phase starvation conditions.
