# Supplementary material for: Molecular Mechanisms of Persistence of Mutualistic Bacteria Photorhabdus in the Entomopathogenic Nematode Host
Source: PLoS One. 2010 Oct 5;5(10):e13154. doi: 10.1371/journal.pone.0013154 (PMC2950140; doi:10.1371/journal.pone.0013154)
Supplement: Table S2 — Oligonucleotide sequences used for quantitative real-time PCR analyses. (0.01 MB PDF) [file pone.0013154.s005.pdf]

Table S2. Oligonucleotide sequences used for quantitative real-time PCR analyses

Gene	Forward primer (5' - 3')	Reverse primer (5' - 3')
fabA	TGAGCCTTGCTACATTGAGTGGGA	AGCCGATGACAGTGAAGGCTACTA
infC	ACCAGCAGATTGGTGTCGAAATGC	TGATCACCTGACGGCCTTCAATCT
iolC	GCACACCCATGATTGGAAACGACT	TGGCATTACCGTACAGGGAGTTCA
menD	TTGCGATCAATCCGTCAATGCCAC	GGCAGTAGCACATCAATTGCACCA
nhaB	TGCGGCCATAAGACAGACGGATTA	GCCGCTATGCAAGAAGGGTTGATT
nuoN	TGCTTTAAACTCTCCCTCGTGCCT	AAGGAACAAGCGCATAACCACAGC
padR	ATGTTCAACTCAAGGCTGCTGTGC	AATGTGTAGACTTTGCGCCCACGA
pbhH	ATGGCAGCCCACATATCCTTGTGA	TCATTGACATTTCCGGCAGTCAGC
pdxY	CCCAATATGCCCAATGGAAAGGGT	ATCCACTCAGAACCGCACTACAAG
pilN	TTGCCGTTGATACAGCGCAATGGT	TTGCCGGGTATACGGCATTTCGAT
proA	TGTGGCAGAAGCAGATTACAGCGA	TAAACCGCCGCAGAATCTACCTGA
purL	AATGCGGCGGCCATTTCTACATGA	GCATCAGGCCAAACGGGATAATCA
tktA	GCACTGCGTTACGGGCATATTCAA	TTGGCATGTCCGCCATTATGAACG
trkA	CCGGTACTTTCTGGGCAACTTTCT	TTCTTTGGCAATCTGGCGACCACT
**16SPt**	AGCGCAACCCTTATCCTTTGTTGC	TCGCGAGGTCGCTTCACTTTGTAT

Note: Primer pairs of 16SPt are used for the reference 16S rRNA gene in *Photorhabdus temperata*.
