# Supplementary material for: Molecular Mechanisms of Persistence of Mutualistic Bacteria Photorhabdus in the Entomopathogenic Nematode Host
Source: PLoS One. 2010 Oct 5;5(10):e13154. doi: 10.1371/journal.pone.0013154 (PMC2950140; doi:10.1371/journal.pone.0013154)
Supplement: Table S3 — Oligonucleotide sequences used to generate Photorhabdus temperata mutant constructs in this study. (0.01 MB PDF) [file pone.0013154.s006.pdf]

Table S3. Oligonucleotide sequences used to generate *Photorhabdus temperata* mutant constructs in this study

***Gene*-Primer**	**Sequence (5' – 3')**
***camR*-P3**	TTGATCGGCACGTAAGAGGT
***camR*-P4**	AATTTCTGCCATTCATCCGC
***dnaK*-P1**	AAAGGGCCCTGTAGCTATTATGGATGGCACGAC
***dnaK*-P2**	ACCTCTTACGTGCCGATCAACAGTTTCGCACGGGTCACT
***dnaK*-P5**	GCGGATGAATGGCAGAAATTAGACCCGTATGCCAATGGTA
***dnaK*-P6**	GCGGGATCCCATCGTCATCTTTCTTCGCACTGT
***ileS*-P1**	AAAGGGCCCGCGCGAACCAAATATGTTAAA
***ileS*-P2**	ACCTCTTACGTGCCGATCAATGACGCCAGCAGCAGGGATAACTA
***ileS*-P5**	GCGGATGAATGGCAGAAATTGGATCTGGAGCCAGCCGAACTGT
***ileS*-P6**	GCGGGATCCCGTTACCGGCAACATTAGTTACAC
***metL*-P1**	AAAGGGCCCGGCCCACGGATCACTAACGGATTA
***metL*-P2**	ACCTCTTACGTGCCGATCAAGCAACTTAAAGATCAGCCGGTAGA
***metL*-P5**	GCGGATGAATGGCAGAAATTCCAGTGCCACCAGCGCCAAACC
***metL*-P6**	GCGGGATCCTCCGCGGCAGGCAGCACGACTAAC
***purL*-P1**	AAAGGGCCCCACCGTGCGAGATACCCTT
***purL*-P2**	ACCTCTTACGTGCCGATCAAACCGATGAAGATAATGCGTTGTTA
***purL*-P5**	GCGGATGAATGGCAGAAATTTCCGGGTTATCGCGTTGAAC
***purL*-P6**	GCGACTAGTGCCAAGAACAGCAACTTCCGGTCA
***tktA*-P1**	AAAGGGCCCGACCACTCGTAAAACCCTTGCTA
***tktA*-P2**	ACCTCTTACGTGCCGATCAATGGCATCCCACTCAGAATAGATTT
***tktA*-P5**	GCGGATGAATGGCAGAAATTCAGTGGTGAGTTACCGGCAAAC
***tktA*-P6**	GCGGGATCCGATAGCGCCATTCATACCGACATA
